# Community Psychiatry Clinic’s Impact on Dementia Care: Insight from Rythm Community Mental Healthcare Program

**DOI:** 10.1002/alz.087497

**Published:** 2025-01-09

**Authors:** M Vaseel, Nalakath A. Uvais

**Affiliations:** ^1^ Iqraa International Hospital and Research Centre, Calicut, Kerala India

## Abstract

**Background:**

Dementia, a major concern in India (1‐10% prevalence), is challenging, particularly in rural areas with a significant treatment gap. Community psychiatry offers a promising solution, aiming to provide essential dementia care in underserved rural India, reflecting innovative approaches to extend mental health services equitably.

**Methods:**

This study, employing a descriptive approach, examines the impact of Rythm community psychiatry clinics, an initiative by IQRAA International Hospital and Research Centre, on dementia care. The analysis focuses on key community activities, volunteer roles, provided services, and the engagement of Community‐Based Organizations (CBOs).

**Results:**

Since August 2022, among the 8 Rythm community clinics with a total of 298 psychiatry patients, 4 clinics specifically address dementia cases. In these 4 clinics, 29 dementia cases were identified in the community, ranging in age from 68 to 95 years, with an average age of 77.5 years. Of the 29 cases, 25 were females, and 4 were males, resulting in a male‐to‐female ratio of 1:6. A post‐retirement group in Kodencherry, a CBO, alone identified 17 out of the total 29 cases, constituting 58% of the total. Nearly 90% of patients are under homecare follow‐up due to mobility restrictions, while the rest utilize clinic‐based consultations. Two rural community clinics also serve tribal patients with dementia. Dementia causes vary, including Alzheimer’s, vascular dementia, and Parkinson’s disease. Targeted case‐finding approaches effectively identify dementia cases early in the community through trained community volunteers, leading to referrals to community clinics and home‐based consultations. Caregiver education, behavioral management, symptomatic relief, psychological services, and specialist referrals contribute to comprehensive community‐level dementia care. Medications such as Donepezil, Memantine, Quetiapine, and Melatonin are employed based on symptom profiles, with Quetiapine being a commonly used antipsychotic. The results indicate a reduction in caregiver burden, fewer hospital admissions, increased access to primary and behavioral care, early case detection, referrals, and improved community awareness, subsequently reducing stigma surrounding dementia.

**Conclusion:**

Community psychiatry clinics significantly impact dementia care by offering targeted case identification, caregiver education, and comprehensive services, ultimately contributing to improved community‐level dementia care and awareness.

